# Poly[bis­(ethanol)(μ_4_-2,3,5,6-tetra­fluoro­benzene-1,4-di­carboxyl­ato)cadmium]

**DOI:** 10.1107/S1600536813026287

**Published:** 2013-10-02

**Authors:** Nakeun Ko, Jaheon Kim

**Affiliations:** a Department of Chemistry, Soongsil University, 369 Sangdo-Ro, Dongjak-Gu, Seoul, 156-743, South Korea

## Abstract

In the title compound, [Cd(C_8_F_4_O_4_)(C_2_H_5_OH)_2_]_*n*_, the Cd^II^ cation sits on an inversion centre and is coordinated by six O atoms from four tetra­fluoro­benzene-1,4-di­carboxyl­ate anions and two ethanol mol­ecules in a distorted octa­hedral geometry. The anionic ligand is also located on an inversion centre, and connects four Cd^II^ cations, generating a two-dimensional polymeric layer parallel to the *ab* plane. Within the layer, the ethanol mol­ecule links F and O atoms of the nearest anionic ligands *via* O—H⋯O and O—H⋯F hydrogen bonds. The ethyl group of the ethanol mol­ecule is disordered over two positions with an occupancy ratio of 0.567 (10):0.433 (10).

## Related literature

For metal-organic frameworks composed of metal ions and 2,3,5,6-tetra­fluoro­benzene-1,4-di­carboxyl­ate (or tetra­fluoro­terephthalate), see: Chen *et al.* (2006 ▶, 2009 ▶); Hulvey, Ayala *et al.* (2009 ▶); Hulvey, Ayala & Cheetham *et al.* (2009 ▶); Hulvey, Falco *et al.* (2009 ▶); Hulvey *et al.* (2011 ▶); Kitaura *et al.* (2004 ▶); MacNeill *et al.* (2011 ▶); Mikhalyova *et al.* (2011 ▶); Seidel *et al.* (2011 ▶); Seidel *et al.* (2012 ▶); Yoon *et al.* (2007 ▶); Yu *et al.* (2011 ▶); Zheng *et al.* (2008 ▶); Zhu *et al.* (2009 ▶).
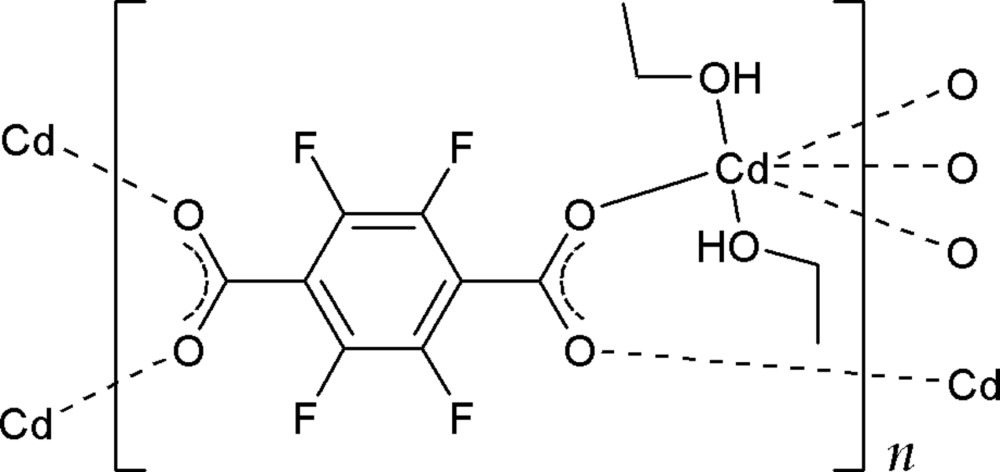


## Experimental

### 

#### Crystal data

[Cd(C_8_F_4_O_4_)(C_2_H_6_O)_2_]
*M* *_r_* = 440.61
Triclinic, 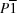

*a* = 4.8367 (3) Å
*b* = 9.0903 (6) Å
*c* = 9.4078 (6) Å
α = 108.091 (1)°
β = 100.637 (1)°
γ = 102.275 (1)°
*V* = 369.95 (4) Å^3^
*Z* = 1
Mo *K*α radiation
μ = 1.55 mm^−1^
*T* = 173 K
0.35 × 0.20 × 0.06 mm

#### Data collection

Bruker SMART APEX CCD diffractometer
Absorption correction: multi-scan (*SADABS*; Bruker, 2001 ▶) *T*_min_ = 0.613, *T*_max_ = 0.913
2308 measured reflections
1576 independent reflections
1569 reflections with *I* > 2σ(*I*)
*R*_int_ = 0.014

#### Refinement

*R*[*F* ^2^ > 2σ(*F* ^2^)] = 0.020
*wR*(*F* ^2^) = 0.054
*S* = 1.11
1576 reflections
131 parameters
6 restraints
H atoms treated by a mixture of independent and constrained refinement
Δρ_max_ = 0.40 e Å^−3^
Δρ_min_ = −0.54 e Å^−3^

### 

Data collection: *SMART* (Bruker, 2007 ▶); cell refinement: *SAINT* (Bruker, 2007 ▶); data reduction: *SAINT*; program(s) used to solve structure: *SHELXTL* (Sheldrick, 2008 ▶); program(s) used to refine structure: *SHELXTL*; molecular graphics: *SHELXTL*; software used to prepare material for publication: *publCIF* (Westrip, 2010 ▶).

## Supplementary Material

Crystal structure: contains datablock(s) global, I. DOI: 10.1107/S1600536813026287/xu5740sup1.cif

Structure factors: contains datablock(s) I. DOI: 10.1107/S1600536813026287/xu5740Isup2.hkl

Additional supplementary materials:  crystallographic information; 3D view; checkCIF report

## Figures and Tables

**Table 1:** Selected bond lengths (Å)

Cd1—O1	2.2526 (15)
Cd1—O2^i^	2.3194 (15)
Cd1—O3	2.2929 (18)

Symmetry code: (i) 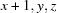.

**Table 2:** Hydrogen-bond geometry (Å, °)

*D*—H⋯*A*	*D*—H	H⋯*A*	*D*⋯*A*	*D*—H⋯*A*
O3—H3*OA*⋯O2^ii^	0.85 (1)	1.94 (2)	2.719 (2)	152 (4)
O3—H3*OB*⋯F2^i^	0.85 (1)	2.40 (2)	3.196 (2)	156 (4)

Symmetry codes: (i) 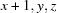; (ii) 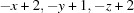.

**Fig. 1.**
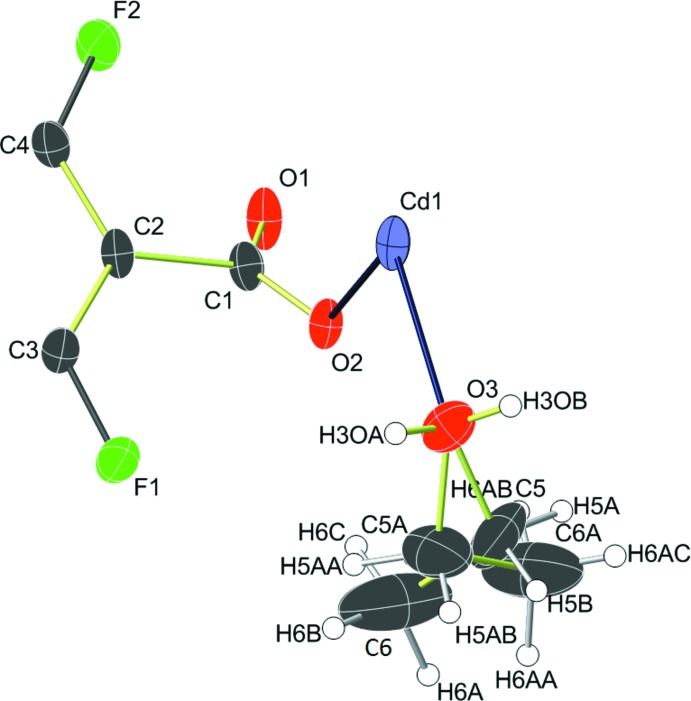
An asymmetric unit of the title compound is shown with the atomic numbering scheme. Displacement ellipsoids are drawn at 50% probability level.

**Fig. 2.**
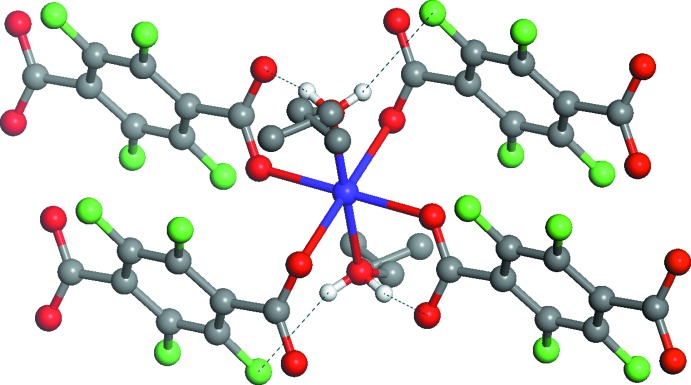
The coordination environment of the title compound is shown with H-bonds (dotted lines).

**Fig. 3.**
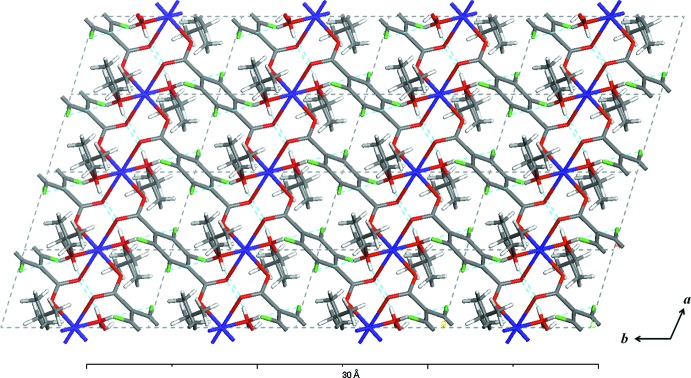
A packing diagram of the title compound is displayed along the c axis. Hydrogen bonds are shown with light blue dotted lines.

**Fig. 4.**
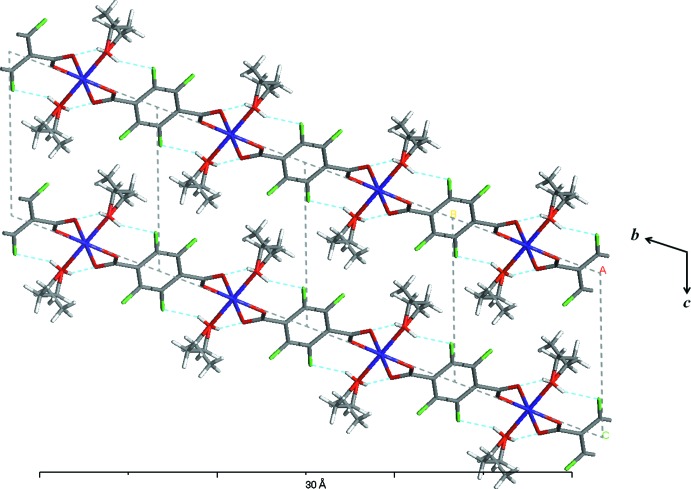
A packing diagram of the title compound is displayed along the a axis. Hydrogen bonds are shown with light blue dotted lines.

## supplementary crystallographic information

### 1. Comment

We reported previously a metal-organic framework (MOF) composed of iron ions and 2,3,5,6-tetrafluorobenzene-1,4-dicarboxylate (or tetrafluoroterephthalate) linkers (Yoon *et al.*, 2007). The title compound in this work was obtained in the course of making a new MOF using cadmium ion with the same organic linker. However, unlike other MOFs prepared through solvothermal reactions in common amine solvent such as *N*,*N*-dimethylformamide, the title compound could be obtained as single crystals in hot ethanol.

### 2. Experimental

The title compound was obtained as colorless plate crystals by a solvothermal reaction between cadmium(II) nitrate tetrahydrate (25 mg) and tetrafluorobenzene-1,4-dicarboxylic acid (12 mg) in ethanol (8 ml)in a Teflon-lined vessel (23 ml) at 353 K and for 2 days.

### 3. Refinement

The ethyl group in ethanol is disordered over two sites with site occupancy factors, 0.56709 (C5 and C6) and 0.43291 (C5A and C6A), respectively. Hydrogen atoms of the ethanol molecule were placed at calculated positions with C—H = 0.99 Å (methylene), C—H = 0.98 Å (methyl) or O—H = 0.85 Å (alcohol) and allowed to ride, with U_iso_(H) = 1.5 U_eq_(C) for methyl H atoms and 1.2U_eq_(C,O) for the others.

### Crystal data

**Table:**

[Cd(C_8_F_4_O_4_)(C_2_H_6_O)_2_]	*Z* = 1
*M**_r_* = 440.61	*F*(000) = 216
Triclinic, *P*1	*D*_x_ = 1.978 Mg m^−^^3^
Hall symbol: -P 1	Mo *K*α radiation, λ = 0.71073 Å
*a* = 4.8367 (3) Å	Cell parameters from 2269 reflections
*b* = 9.0903 (6) Å	θ = 2.4–28.2°
*c* = 9.4078 (6) Å	µ = 1.55 mm^−^^1^
α = 108.091 (1)°	*T* = 173 K
β = 100.637 (1)°	Plate, colorless
γ = 102.275 (1)°	0.35 × 0.20 × 0.06 mm
*V* = 369.95 (4) Å^3^	

### Data collection

**Table:**

Bruker SMART APEX CCD diffractometer	1576 independent reflections
Radiation source: fine-focus sealed tube	1569 reflections with *I* > 2σ(*I*)
Graphite monochromator	*R*_int_ = 0.014
phi and ω scans	θ_max_ = 27.1°, θ_min_ = 2.4°
Absorption correction: multi-scan (*SADABS*; Bruker, 2001)	*h* = −5→6
*T*_min_ = 0.613, *T*_max_ = 0.913	*k* = −11→11
2308 measured reflections	*l* = −12→8

### Refinement

**Table:**

Refinement on *F*^2^	6 restraints
Least-squares matrix: full	H atoms treated by a mixture of independent and constrained refinement
*R*[*F*^2^ > 2σ(*F*^2^)] = 0.020	*w* = 1/[σ^2^(*F*_o_^2^) + (0.0317*P*)^2^ + 0.1686*P*] where *P* = (*F*_o_^2^ + 2*F*_c_^2^)/3
*wR*(*F*^2^) = 0.054	(Δ/σ)_max_ < 0.001
*S* = 1.11	Δρ_max_ = 0.40 e Å^−^^3^
1576 reflections	Δρ_min_ = −0.54 e Å^−^^3^
131 parameters	

### Special details

**Table:**

Geometry. Geometry. All e.s.d.'s (except the e.s.d. in the dihedral angle between two l.s. planes) are estimated using the full covariance matrix. The cell e.s.d.'s are taken into account individually in the estimation of e.s.d.'s in distances, angles and torsion angles; correlations between e.s.d.'s in cell parameters are only used when they are defined by crystal symmetry. An approximate (isotropic) treatment of cell e.s.d.'s is used for estimating e.s.d.'s involving l.s. planes.Refinement. Refinement of F^2^ against ALL reflections. The weighted *R*-factor *wR* and goodness of fit S are based on F^2^, conventional *R*-factors *R* are based on F, with F set to zero for negative F^2^. The threshold expression of F^2^ > 2sigma(F^2^) is used only for calculating *R*-factors(gt) *etc*. and is not relevant to the choice of reflections for refinement. *R*-factors based on F^2^ are statistically about twice as large as those based on F, and R– factors based on ALL data will be even larger.

### Fractional atomic coordinates and isotropic or equivalent isotropic displacement parameters (Å^2^)

**Table:**

	*x*	*y*	*z*	*U*_iso_*/*U*_eq_	Occ. (<1)
Cd1	1.0000	0.5000	1.0000	0.02063 (9)	
F1	0.1389 (4)	0.24768 (17)	1.27515 (16)	0.0358 (3)	
F2	0.2660 (3)	0.01945 (17)	0.77434 (17)	0.0349 (3)	
O1	0.6687 (3)	0.28942 (19)	1.0084 (2)	0.0271 (3)	
O2	0.3899 (3)	0.43185 (17)	1.12102 (18)	0.0227 (3)	
C1	0.4446 (4)	0.3017 (2)	1.0543 (2)	0.0205 (4)	
C2	0.2191 (4)	0.1450 (2)	1.0267 (2)	0.0194 (4)	
C3	0.0738 (5)	0.1268 (3)	1.1375 (2)	0.0226 (4)	
C4	0.1397 (5)	0.0137 (3)	0.8888 (3)	0.0219 (4)	
O3	1.0823 (4)	0.3384 (2)	0.7802 (2)	0.0327 (4)	
H3OA	1.249 (5)	0.393 (5)	0.781 (5)	0.039*	0.567 (10)
H3OB	1.137 (15)	0.267 (5)	0.809 (5)	0.039*	0.433 (10)
C5	0.8671 (16)	0.2366 (7)	0.6350 (7)	0.055 (2)	0.567 (10)
H5A	0.6914	0.1769	0.6553	0.065*	0.567 (10)
H5B	0.9502	0.1568	0.5726	0.065*	0.567 (10)
C6	0.783 (2)	0.3392 (10)	0.5484 (8)	0.094 (4)	0.567 (10)
H6A	0.6335	0.2711	0.4509	0.141*	0.567 (10)
H6B	0.9566	0.3951	0.5257	0.141*	0.567 (10)
H6C	0.7039	0.4190	0.6115	0.141*	0.567 (10)
C5A	0.9372 (18)	0.3012 (15)	0.6188 (7)	0.064 (3)	0.433 (10)
H5AA	0.9215	0.4015	0.6017	0.076*	0.433 (10)
H5AB	1.0524	0.2508	0.5514	0.076*	0.433 (10)
C6A	0.6405 (16)	0.1883 (14)	0.5801 (9)	0.077 (4)	0.433 (10)
H6AA	0.5410	0.1599	0.4705	0.116*	0.433 (10)
H6AB	0.5261	0.2403	0.6452	0.116*	0.433 (10)
H6AC	0.6579	0.0902	0.5990	0.116*	0.433 (10)

### Atomic displacement parameters (Å^2^)

**Table:**

	*U*^11^	*U*^22^	*U*^33^	*U*^12^	*U*^13^	*U*^23^
Cd1	0.01309 (12)	0.01499 (12)	0.03701 (14)	0.00397 (8)	0.01226 (8)	0.01066 (9)
F1	0.0477 (9)	0.0235 (7)	0.0292 (7)	−0.0016 (6)	0.0166 (6)	0.0042 (6)
F2	0.0434 (8)	0.0268 (7)	0.0387 (7)	0.0039 (6)	0.0284 (6)	0.0117 (6)
O1	0.0160 (7)	0.0204 (8)	0.0512 (10)	0.0062 (6)	0.0155 (7)	0.0174 (7)
O2	0.0184 (7)	0.0165 (7)	0.0331 (8)	0.0035 (6)	0.0083 (6)	0.0094 (6)
C1	0.0137 (9)	0.0186 (10)	0.0310 (10)	0.0029 (7)	0.0061 (7)	0.0127 (8)
C2	0.0128 (8)	0.0175 (9)	0.0320 (10)	0.0050 (7)	0.0081 (7)	0.0131 (8)
C3	0.0214 (10)	0.0188 (10)	0.0273 (10)	0.0045 (8)	0.0079 (8)	0.0079 (8)
C4	0.0196 (10)	0.0219 (10)	0.0302 (10)	0.0063 (8)	0.0140 (8)	0.0132 (8)
O3	0.0258 (8)	0.0301 (9)	0.0332 (9)	0.0046 (7)	0.0055 (7)	0.0032 (7)
C5	0.051 (5)	0.033 (3)	0.049 (3)	−0.001 (3)	−0.009 (3)	−0.005 (3)
C6	0.109 (7)	0.128 (8)	0.040 (4)	0.079 (7)	0.002 (4)	0.004 (4)
C5A	0.053 (5)	0.072 (7)	0.033 (4)	−0.028 (5)	−0.004 (3)	0.015 (4)
C6A	0.033 (4)	0.124 (9)	0.036 (4)	−0.017 (5)	0.000 (3)	0.009 (4)

### Geometric parameters (Å, º)

**Table:**

Cd1—O1^i^	2.2526 (15)	O3—C5	1.444 (4)
Cd1—O1	2.2526 (15)	O3—C5A	1.449 (5)
Cd1—O2^ii^	2.3194 (15)	O3—H3OA	0.850 (5)
Cd1—O2^iii^	2.3194 (15)	O3—H3OB	0.849 (5)
Cd1—O3^i^	2.2929 (18)	C5—C6	1.487 (5)
Cd1—O3	2.2929 (18)	C5—H5A	0.9900
F1—C3	1.343 (3)	C5—H5B	0.9900
F2—C4	1.342 (2)	C6—H6A	0.9800
O1—C1	1.252 (3)	C6—H6B	0.9800
O2—C1	1.264 (3)	C6—H6C	0.9800
O2—Cd1^iv^	2.3194 (15)	C5A—C6A	1.481 (5)
C1—C2	1.513 (3)	C5A—H5AA	0.9900
C2—C4	1.384 (3)	C5A—H5AB	0.9900
C2—C3	1.391 (3)	C6A—H6AA	0.9800
C3—C4^v^	1.382 (3)	C6A—H6AB	0.9800
C4—C3^v^	1.382 (3)	C6A—H6AC	0.9800
			
O1^i^—Cd1—O1	180.0	C5—O3—H3OA	120 (3)
O1^i^—Cd1—O3^i^	91.52 (7)	C5A—O3—H3OA	97 (3)
O1—Cd1—O3^i^	88.48 (6)	Cd1—O3—H3OA	103 (3)
O1^i^—Cd1—O3	88.48 (6)	C5—O3—H3OB	100 (4)
O1—Cd1—O3	91.52 (7)	C5A—O3—H3OB	120 (3)
O3^i^—Cd1—O3	180.0	Cd1—O3—H3OB	103 (3)
O1^i^—Cd1—O2^ii^	87.93 (6)	H3OA—O3—H3OB	98 (6)
O1—Cd1—O2^ii^	92.07 (6)	O3—C5—C6	109.1 (5)
O3^i^—Cd1—O2^ii^	97.63 (6)	O3—C5—H5A	109.9
O3—Cd1—O2^ii^	82.37 (6)	C6—C5—H5A	109.9
O1^i^—Cd1—O2^iii^	92.07 (5)	O3—C5—H5B	109.9
O1—Cd1—O2^iii^	87.93 (6)	C6—C5—H5B	109.9
O3^i^—Cd1—O2^iii^	82.37 (6)	H5A—C5—H5B	108.3
O3—Cd1—O2^iii^	97.63 (6)	C5—C6—H6A	109.5
O2^ii^—Cd1—O2^iii^	180.0	C5—C6—H6B	109.5
C1—O1—Cd1	123.39 (14)	H6A—C6—H6B	109.5
C1—O2—Cd1^iv^	120.48 (13)	C5—C6—H6C	109.5
O1—C1—O2	126.19 (19)	H6A—C6—H6C	109.5
O1—C1—C2	116.46 (19)	H6B—C6—H6C	109.5
O2—C1—C2	117.36 (18)	C6A—C5A—O3	108.5 (5)
C4—C2—C3	116.23 (19)	C6A—C5A—H5AA	110.0
C4—C2—C1	122.13 (19)	O3—C5A—H5AA	110.0
C3—C2—C1	121.62 (19)	C6A—C5A—H5AB	110.0
F1—C3—C4^v^	117.7 (2)	O3—C5A—H5AB	110.0
F1—C3—C2	120.24 (19)	H5AA—C5A—H5AB	108.4
C4^v^—C3—C2	122.0 (2)	C5A—C6A—H6AA	109.5
F2—C4—C3^v^	117.5 (2)	C5A—C6A—H6AB	109.5
F2—C4—C2	120.81 (19)	H6AA—C6A—H6AB	109.5
C3^v^—C4—C2	121.7 (2)	C5A—C6A—H6AC	109.5
C5—O3—Cd1	127.3 (4)	H6AA—C6A—H6AC	109.5
C5A—O3—Cd1	129.2 (6)	H6AB—C6A—H6AC	109.5
			
Cd1—O1—C1—O2	−10.2 (3)	C4—C2—C3—C4^v^	−0.6 (3)
Cd1—O1—C1—C2	169.78 (13)	C1—C2—C3—C4^v^	177.71 (19)
Cd1^iv^—O2—C1—O1	113.9 (2)	C3—C2—C4—F2	179.70 (19)
Cd1^iv^—O2—C1—C2	−66.1 (2)	C1—C2—C4—F2	1.4 (3)
O1—C1—C2—C4	−41.9 (3)	C3—C2—C4—C3^v^	0.6 (3)
O2—C1—C2—C4	138.0 (2)	C1—C2—C4—C3^v^	−177.70 (19)
O1—C1—C2—C3	139.9 (2)	C5A—O3—C5—C6	−28.4 (13)
O2—C1—C2—C3	−40.1 (3)	Cd1—O3—C5—C6	76.5 (7)
C4—C2—C3—F1	179.42 (19)	C5—O3—C5A—C6A	23.6 (10)
C1—C2—C3—F1	−2.3 (3)	Cd1—O3—C5A—C6A	−73.7 (11)

Symmetry codes: (i) −*x*+2, −*y*+1, −*z*+2; (ii) *x*+1, *y*, *z*; (iii) −*x*+1, −*y*+1, −*z*+2; (iv) *x*−1, *y*, *z*; (v) −*x*, −*y*, −*z*+2.

### Hydrogen-bond geometry (Å, º)

**Table:**

*D*—H···*A*	*D*—H	H···*A*	*D*···*A*	*D*—H···*A*
O3—H3*OA*···O2^i^	0.85 (1)	1.94 (2)	2.719 (2)	152 (4)
O3—H3*OB*···F2^ii^	0.85 (1)	2.40 (2)	3.196 (2)	156 (4)

Symmetry codes: (i) −*x*+2, −*y*+1, −*z*+2; (ii) *x*+1, *y*, *z*.
